# Structural and Thermal Effects of Beeswax Incorporation in Electrospun PVA Nanofibers

**DOI:** 10.3390/ma18143293

**Published:** 2025-07-12

**Authors:** Margarita P. Neznakomova, Fabien Salaün, Peter D. Dineff, Tsvetozar D. Tsanev, Dilyana N. Gospodinova

**Affiliations:** 1 Faculty of Industrial Technology, Technical University of Sofia, 1756 Sofia, Bulgaria; mneznakomova@tu-sofia.bg; 2 ULR 2461-GEMTEX-Génie et Matériaux Textiles, Ecole Nationale Supérieure des Arts et Industries Textiles, Université de Lille, F-59000 Lille, France; fabien.salaun@ensait.fr; 3 Faculty of Electrical Engineering, Technical University of Sofia, 1756 Sofia, Bulgaria; dineff_pd@abv.bg; 4 Faculty of Electronic Engineering and Technologies, Technical University of Sofia, 1756 Sofia, Bulgaria; zartsanev@tu-sofia.bg

**Keywords:** electrospinning, polyvinyl alcohol, beeswax, nanofibers, thermal stability, wound dressing, biodegradation

## Abstract

This study presents the development and characterization of electrospun nanofibers composed of polyvinyl alcohol (PVA) and natural beeswax (BW). A stable emulsion containing 9 wt% PVA and 5 wt% BW was successfully formulated and electrospun. The effects of beeswax incorporation on solution properties-viscosity, conductivity, and surface tension—were systematically evaluated. Electrospinning was performed at 30 kV and a working distance of 14.5 cm, yielding nanofibers with diameters between 125 and 425 nm. Scanning electron microscopy (SEM) revealed increased surface roughness and diameter variability in PVA/BW fibers compared to the PVA. Fourier transform infrared spectroscopy (FTIR) confirmed physical incorporation of BW without evidence of chemical bonding. Thermogravimetric and differential scanning calorimetry analyses (TGA/DSC) demonstrated altered behavior and an expanded profile of temperature transitions due to the waxy components. The solubility test of the nanofiber mat in saline indicated that BW slows dissolution and improves the structural integrity of the fibers. This study demonstrates, for the first time, the incorporation of beeswax into electrospun PVA nanofibers with improved structural and thermal properties, indicating potential for further exploration in biomedical material design.

## 1. Introduction

The production of chemical fibers and fibrous structures encompasses a variety of methods, among which electrospinning is particularly notable for its simplicity, energy efficiency, and versatility [1,2,3]. This technique enables the fabrication of non-woven nanofibers with diameters ranging from tens of nanometers to micrometers, offering high surface area, tunable porosity, and structural similarity to the extracellular matrix, features highly valued in biomedical applications [4,5,6,7,8,9]. The electrospinning process is governed by several interdependent factors, such as polymer concentration, viscosity, surface tension, conductivity, applied voltage, collector distance, and ambient humidity [10,11,12].

Among commonly used polymers, polyvinyl alcohol (PVA) stands out due to its water solubility, biocompatibility, and ease of processing [12,13,14,15]. Its abundant hydroxyl groups contribute to hydrophilicity, enhancing biological interaction but simultaneously reducing structural stability in aqueous media [16,17]. To overcome this limitation, researchers have explored blending PVA with hydrophobic natural additives, aiming to improve water resistance and broaden application potential [18,19]. Previous studies have also explored electrospinning of beeswax blended with poly(ethylene oxide) (PEO), demonstrating the feasibility of incorporating beeswax into polymer fibers and highlighting its antimicrobial and release-modifying properties, despite differences in solvent systems [20,21].

Beeswax (BW) is a natural, biodegradable wax composed of esters, hydrocarbons, and fatty acids [22,23], long used in medical and cosmetic formulations for its antibacterial, anti-inflammatory, and moisture-repellent properties [24,25,26,27]. Owing to its hydrophobicity and thermal behavior, BW holds promise as a functional modifier in polymer systems [28,29]. However, its incorporation into hydrophilic matrices like PVA presents significant challenges due to low water solubility and incompatibility at the molecular level [30,31,32]. Recent efforts have focused on emulsification strategies that stabilize BW in aqueous media, typically as oil-in-water systems, depending on droplet size and crystalline structure [33,34].

Previous studies have shown that blending BW with polymers such as polycaprolactone (PCL) or polylactic acid (PLA) can significantly alter fiber morphology, surface wettability, and porosity. For instance, BW-modified PCL fibers exhibit increased surface roughness and hydrophobicity [35,36], while BW-containing PLA fibers have demonstrated thermal healing, anti-adhesive behavior, and even bacteriostatic activity in peritendinous applications [37,38,39,40].

Despite these advances, the integration of BW into PVA-based electrospun systems remains underexplored, particularly with regard to its physicochemical and biofunctional effects. Most available studies focus on surface modification rather than fiber structure or regenerative properties [41]. Moreover, the antibacterial efficacy of BW may be enhanced through purification especially water-based methods which remove impurities and concentrate bioactive compounds such as flavonoids and residual propolis [42].

Given the known biocompatibility of PVA and the functional properties of beeswax, this study aims to develop and characterize electrospun PVA/BW nanofiber mats for potential biomedical applications. The novelty lies in the stabilization and integration of a hydrophobic natural additive (BW) into a hydrophilic polymer (PVA) through emulsification, without the need for chemical modification. To address these limitations and knowledge gaps, this study investigates the integration of natural beeswax into PVA-based electrospun nanofibers, aiming to enhance their physicochemical and functional properties for biomedical use.

The specific objectives of this study are the following:

- Develop a stable microemulsion of PVA and BW suitable for electrospinning;
- Identify optimal electrospinning parameters based on solution properties and fiber formation [4,5,6];
- Characterize the resulting nanofibers in terms of morphology (SEM), chemical structure (FTIR), and thermal behavior (TGA/DSC) [10,23,24,36];
- Evaluate degradation behavior of the fibers in physiological saline solution, simulating biomedical exposure [25,26,27,28];
- Assess the impact of textile-based collector surfaces on fiber deposition, uniformity, and mat structure [40,41].

A key strength of this study lies in the successful stabilization and electrospinning of a PVA/BW microemulsion, overcoming the inherent incompatibility of hydrophilic and hydrophobic phases without the need for chemical modification.

The skin-mimicking textile collector marks a practical innovation, enhancing the clinical relevance of the fiber deposition process.

Enhanced thermal stability may improve not only processing but also the performance of these mats under storage, sterilization, or in thermally variable environments.

Through this study, we aim to provide a biodegradable, bioactive nanofiber system composed of natural and synthetic components that demonstrates improved stability, thermal performance, and potential regenerative properties, with direct applicability in wound care and dermal therapy.

However, while increased hydrophobicity may improve breathability and moisture barrier function, it may also reduce hydration at the wound interface. This trade-off should be carefully considered when designing bioactive wound dressings, where both fluid management and tissue moisture are essential for healing.

## 2. Materials and Methods

### 2.1. Materials

Polyvinyl alcohol (PVA) was purchased from Merck KGaA (Darmstadt, Germany) with a molecular weight of 79,000 g·mol^−1^ and a degree of hydrolysis of 92.5 mol%. Natural beeswax (BW) was obtained from *Apis mellifera ligustica* hives in northwestern Bulgaria (Agri BG). The beeswax had a melting point range of 62–67 °C, a melting enthalpy of 162.4 J·g^−1^, a saponification value of 95 mg·g^−1^, and an acid value of 12 mg·g^−1^. Before use, the wax was purified by melting in water at 70–75 °C to enhance its antibacterial properties. [43,44], resulting in a purified wax with a relative density of 0.96. Deionized water (conductivity 1 μS·cm^−1^) was used to prepare all solutions.

The natural beeswax used in this study was purified prior to use through aqueous heating and decantation, following established procedures commonly applied in cosmetic-grade wax preparation. The chemical composition of the purified beeswax was not analyzed in detail; however, its melting point, enthalpy, acid, and saponification values are consistent with standardized values reported for natural beeswax from Apis mellifera.

### 2.2. Preparation of Polymer Solutions

Heptane was used as a transient co-solvent to facilitate the dispersion of molten beeswax. During high-speed homogenization at 95 °C, heptane evaporated completely, resulting in a stable oil-in-water (O/W) emulsion of BW in aqueous medium. The final electrospinning solution contained only water, PVA, and emulsified beeswax droplets.

To enhance the dispersibility of natural beeswax (BW) in the aqueous PVA solution, a two-step emulsification procedure was used. First, the beeswax was cut into small pieces and melted at 70 °C under constant stirring (400 rpm). Heptane was added in a 2:1 weight ratio (BW:heptane) to assist in the initial dispersion of the hydrophobic wax. This intermediate mixture was gradually combined with deionized water and homogenized at 10,000 rpm for 10 min to form a pre-emulsion.

The emulsion was then heated to 95 °C for 10 min under high-speed homogenization at 12,000 rpm. Under these conditions, heptane, being highly volatile (boiling point ≈ 98 °C), evaporated completely, leaving behind a stable oil-in-water (O/W) emulsion of beeswax droplets dispersed in aqueous medium. The final emulsion was cooled to room temperature and filtered through a 0.45 µm membrane to remove any residual wax aggregates.

**Figure 1:**
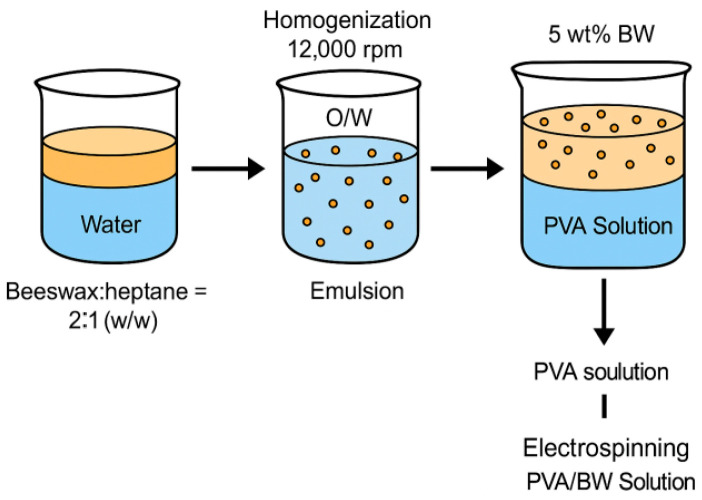
Schematic illustration of the preparation of the electrospinning solution: beeswax–heptane mixture was emulsified in water, then combined with PVA solution to form a 5 wt% BW/PVA mixture used for electrospinning.

The beeswax content was adjusted to 5 wt% relative to the dry weight of PVA, and the resulting emulsion was directly mixed with 9 wt% PVA solution to prepare the final electrospinning mixture. The emulsion showed no visible phase separation after 24 h of storage, indicating sufficient stability for subsequent fiber formation. A schematic representation of this process is shown in Figure 1.

### 2.3. Preparation of Beeswax Microemulsion

To enhance dispersibility, beeswax was cut into small pieces and melted at 70 °C under continuous stirring at 400 rpm. Heptane was added in a 2:1 ratio (wax: heptane), followed by the slow addition of deionized water. The mixture was homogenized at 10,000 rpm for 10 min. The emulsion was left undisturbed at 23 °C for 22–24 h and filtered to remove residual solid particles.

The final PVA/BW mixture appeared visually stable and homogeneous, with no signs of phase separation for at least 24 h. Although no direct microscopic characterization of the droplet–polymer interactions was conducted, the system behaved as a stable oil-in-water dispersion, suitable for electrospinning.

### 2.4. Electrospinning Procedure

Electrospinning was carried out using a laboratory-scale electrospinning apparatus consisting of a high-voltage power supply, a syringe pump, a stainless-steel blunt-tip needle (23G for PVA and 27G for PVA/BW), and a grounded collector plate. The polymer solution was loaded into a 5 mL plastic syringe connected to the needle via a flexible Teflon tube. The solution feed rate was controlled using a precision syringe pump.

For the PVA solution (9 wt%), the optimum electrospinning parameters were determined to be a voltage of 30 kV, a needle-to-collector distance of 18 cm, and a flow rate of 1 μL·min^−1^. For the PVA/BW solution (9 wt% PVA + 5 wt% BW), the optimum parameters included the same applied voltage (30 kV), a reduced needle-to-collector distance of 14.5 cm, and the same flow rate (1 μL·min^−1^). The electrospinning time for both solutions was set to 90 min.

The textile fabrics used as a collector substrate was a fabric made of 100% cotton, exhibits strong moisture absorption and management properties due to its fiber composition and construction. The thickness and weight of the selected carrier for the electrospun nanofibers affect how effectively moisture transfer occurs when they are spun. The selected fabric is a twill weave, dyed in indigo, with the following characteristics: surface weight 180 g/m^2^, and thickness 0.87 mm. The goal was to replace the typical smooth surface of the collector with a rough substrate that would attract and retain electrospun fibers. The woven structure of the textile fabric was expected to affect the local electric field.

### 2.5. Solution Characterization

Viscosity was measured at room temperature (20 ± 0.15 °C) using a SMART Series digital rotational viscometer (Fungilab, Barcelona, Spain). Conductivity was assessed with a DDS-1702 conductivity meter (Shanghai BOQU Instruments, Shanghai, China), and pH was measured using an AD14 pH/ORT Tester (ADWA Instruments, Szeged, Hungary). All measurements were repeated five times.

All samples were cut into uniform discs with a diameter of 20 mm and weighed before testing (W_0_). The nanofiber mats were submerged in 10 mL of 0.9% NaCl solution (physiological saline) and incubated at 36 ± 1 °C for up to 120 min. The degradation experiments were performed in sealed containers under static conditions, without agitation, to minimize evaporation.

At predefined time intervals (30, 60, 90, and 120 min), samples were gently removed, briefly rinsed with distilled water to eliminate surface salt, and dried at ambient temperature before reweighing (W_t_). The percentage of mass loss was calculated using Equation (1):

Mass loss = ((W_0_ − W_t_)/W_0_) × 100, % (1)

At least n = 5 independently prepared fiber mats were tested per group, and results are reported as mean ± standard deviation (SD).

### 2.6. Morphological and Structural Characterization

The morphology of the electrospun nanofibers was evaluated using scanning electron microscopy (SEM). Samples were sputter-coated with carbon to ensure conductivity and imaged using a Hitachi SU5000 SEM (Tokyo, Japan) operating at an accelerating voltage of 4 kV. Fiber diameters were measured from SEM micrographs using ImageJ software (version 1.54 g, NIH, Bethesda, MD, USA). Images were first calibrated using the embedded scale bars, and measurements were taken using the software’s line tool with pixel-to-micron conversion applied. For each sample type, at least 300 fibers were randomly selected across multiple regions of the mat to ensure statistical robustness.

Confocal laser scanning microscopy (CLSM) was employed to assess the thickness of the nanofiber layers deposited on the textile substrate. A KEYENCE VK 9710K confocal microscope (Osaka, Japan) was used for topographic imaging and surface profiling.

The chemical structure of the electrospun mats was analyzed using Fourier-transform infrared spectroscopy (FTIR). For FTIR analysis, electrospun fiber mats were cut into circular samples (∅ 20 mm), air-dried for 48 h, then vacuum-dried at 40 °C for 12 h to reduce residual moisture. Spectra were recorded using a Thermo Scientific Nicolet iS50 spectrometer (Waltham, MA, USA) with an ATR crystal, scanning from 4000 to 650 cm^−1^, with a resolution of 2 cm^−1^ and 64 averaged scans per sample.

Thermal behavior was evaluated by thermogravimetric analysis (TGA) and differential scanning calorimetry (DSC). Analyses were performed using a Mettler Toledo TGA/DSC 3+ system (Columbus, OH, USA) under a nitrogen atmosphere (flow rate: 50 mL·min^−1^). Approximately 10 mg of sample was used for each test. The temperature range was set from 20 to 600 °C with a heating rate of 10 °C·min^−1^.

### 2.7. Statistical Analysis

All statistical analyses were performed using OriginPro2023 (academic). A significance level of *p* < 0.05 was considered statistically significant. All values are reported as mean ± standard deviation (SD) unless otherwise noted.

## 3. Results and Discussion

### 3.1. Electrospinning Solution Properties

The fiber-forming ability of a polymer solution is strongly dependent on its rheological and physicochemical properties, particularly viscosity, electrical conductivity, and surface tension. These parameters influence the formation and stability of the electrospinning jet, the morphology of the ultrafine polymer fibers, and the occurrence of defects such as beads or droplets.

**Figure 2:**
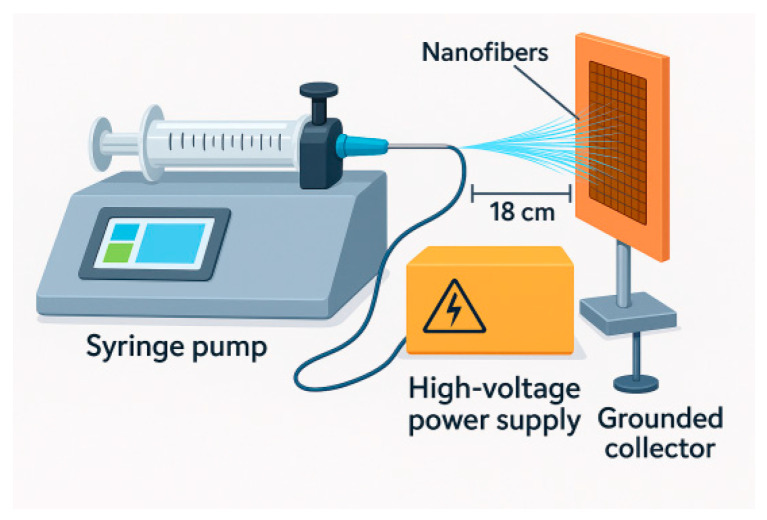
Schematic representation of the electrospinning setup.

In this study, the electrospinning behavior of two solutions was evaluated: a 9 wt% PVA solution and a composite solution with 9 wt% PVA and 5 wt% beeswax (BW). The PVA solution exhibited favorable electrospinnability at the selected PVA concentration, with a viscosity of 0.233 Pa·s, electrical conductivity of 0.29 μS·cm^−1^, and surface tension appropriate for producing smooth, continuous nanofibers. These values fall within the optimal ranges reported for defect-free nanofiber production [45,46]. Figure 2 shows the dependence of these key parameters on PVA concentration, demonstrating that viscosity and conductivity increase nonlinearly, while surface tension exhibits a more gradual, linear increase.

**Figure 3:**
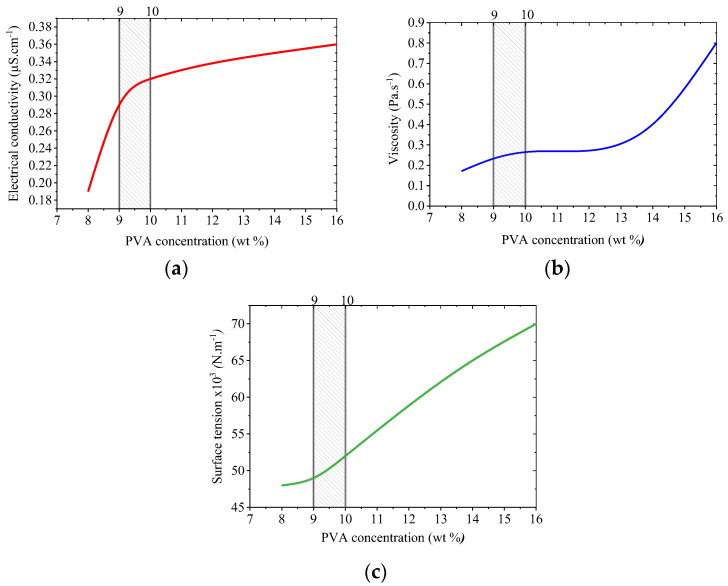
Effect of PVA concentration on selected physicochemical parameters of aqueous solutions relevant to electrospinning: electrical conductivity (**a**), dynamic viscosity (**b**), and surface tension (**c**).

Among the tested concentrations, 9 wt% PVA showed a favorable balance of viscosity (Figure 3b), conductivity (Figure 3a), and surface tension (Figure 3c), supporting efficient electrospinning. The viscosity at this level was high enough to ensure chain entanglement, while the conductivity and surface tension remained within acceptable limits, enabling uniform fiber formation without bead defects.

**Figure 4:**
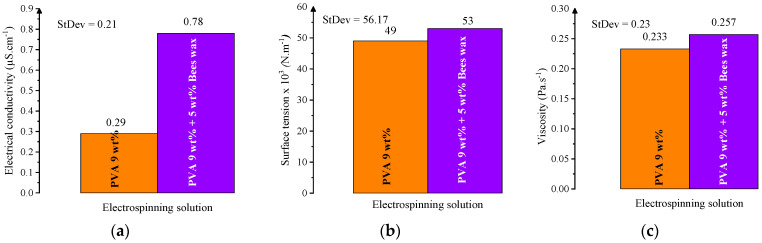
Effect of beeswax (BW) addition (5 wt%) on the physicochemical properties of 9 wt% PVA solution: (**a**) electrical conductivity, (**b**) surface tension, (**c**) viscosity.

Rheological analysis confirmed that both solutions exhibited non-Newtonian, shear-thinning behavior, typical for polymeric systems. The inclusion of BW led to oscillations in viscosity values at low shear rates, suggesting microstructural instability or emulsion heterogeneity under minimal deformation. Such irregularities may contribute to slight fiber diameter variability or the appearance of occasional morphological defects during electrospinning (Figure 4).

Overall, the addition of beeswax necessitated a refinement of electrospinning conditions, particularly the reduction in the needle-to-collector distance, to compensate for the altered balance of forces within the spinning jet. Nevertheless, the PVA/BW solution remained electrospinnable, demonstrating the feasibility of incorporating natural waxes into water-based nanofiber systems.

**Figure 5:**
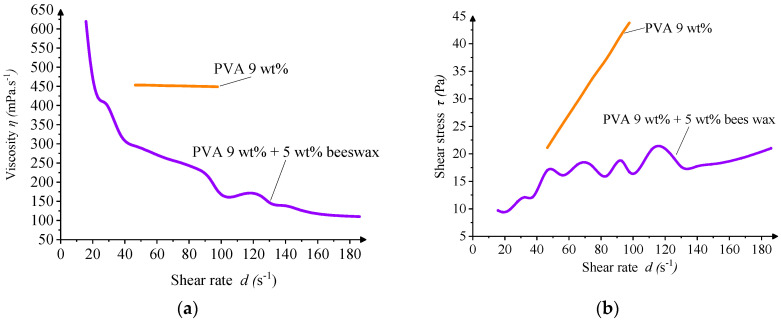
Rheological behavior of electrospinning solutions: viscosity versus shear rate for (**a**) 9 wt% PVA and (**b**) 9 wt% PVA + 5 wt% BW.

The addition of beeswax significantly altered the physicochemical profile of the solution. The PVA/BW emulsion demonstrated increased viscosity and surface tension, while its conductivity was slightly reduced compared to the PVA solution. These results are presented in Figure 5, which compares the three key parameters for both PVA and PVA/BW systems. The presence of BW influences intermolecular interactions and alters fluidity due to its hydrophobic nature and lubricating effect.

The wave-like undulations observed in the viscosity profile of the PVA/BW emulsion may arise from dynamic interactions between wax droplets under shear, including rearrangement or transient coalescence. Such effects are not uncommon in complex emulsions and may be amplified by instrumental sensitivity at lower shear rates. Nevertheless, the solution retains overall pseudoplastic (shear-thinning) behavior suitable for electrospinning.

### 3.2. Nanofiber Morphology and Structure

#### 3.2.1. SEM Analysis

**Figure 6:**
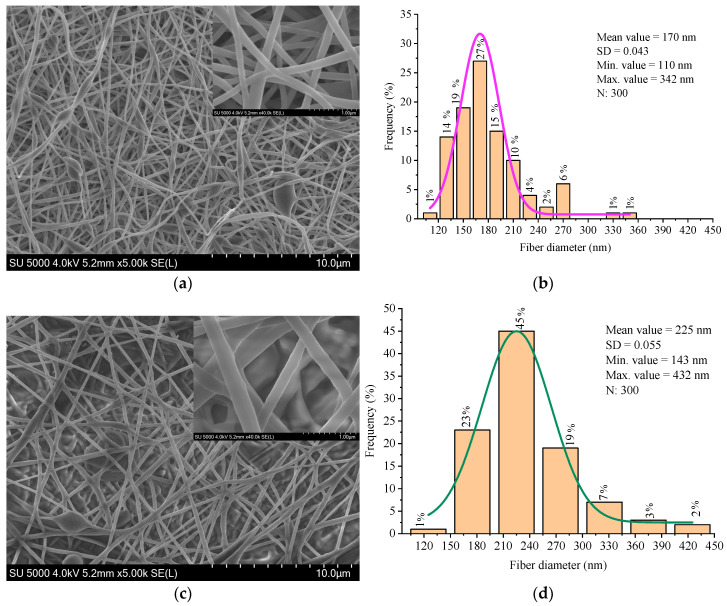
Morphology and diameter distribution of electrospun nanofibers: (**a**) SEM image of 9 wt% PVA fibers; (**b**) diameter distribution histogram of PVA fibers; (**c**) SEM image of 9 wt% PVA + 5 wt% BW fibers; (**d**) diameter distribution histogram of PVA/BW fibers.

The surface morphology and microstructure of the electrospun nanofibers were investigated using scanning electron microscopy (SEM). The fibers obtained from the 9 wt% PVA solution exhibited a relatively smooth and uniform morphology, with a mean diameter of approximately 170 nm. Occasional spindle-shaped beads were observed along the fiber axis, which are typically associated with subcritical viscosity or transient instability in the electrospinning jet. Figure 6 shows the SEM micrograph of the PVA nanofibers, where these features are visible.

The fiber diameters were measured from the SEM images using ImageJ software. The statistical robustness was ensured by 300 measurements randomly taken across different fields [47].

The nanofibers produced from the PVA/BW emulsion exhibited increased average diameter, broader diameter distribution, and noticeably rougher surface texture compared to pure PVA fibers (Figure 6). Occasional fiber merging and localized thickening were observed, likely due to the altered rheological properties and microstructural instability of the emulsion. These morphological changes are attributed to partial migration of beeswax to the fiber surface during jet elongation and solvent evaporation, which affects surface energy and promotes fiber-to-fiber adhesion. Similar effects have been reported in other wax- or lipid-modified electrospun systems, where surface-active components reorganize during drying and influence fiber morphology and interfacial behavior [48,49,50].

The PVA nanofibers showed a narrower diameter distribution, while the PVA/BW fibers exhibited a broader distribution, indicating lower uniformity in fiber formation. These results confirm that beeswax significantly affects fiber formation, increasing average diameter and surface roughness, probably due to its non-uniform dispersion and impact on solution viscosity and jet dynamics. Although the composite fibers exhibit some structural irregularities, the morphology remains within acceptable parameters for biomedical nanofiber mats.

Table 1 summarizes the fiber diameter parameters obtained from SEM analysis of electrospun mats produced from PVA and PVA/beeswax (BW) solutions. The incorporation of BW led to a noticeable increase in the average fiber diameter and standard deviation, indicating a broader diameter distribution and reduced uniformity in fiber morphology.

#### 3.2.2. FTIR Analysis

**Figure 7:**
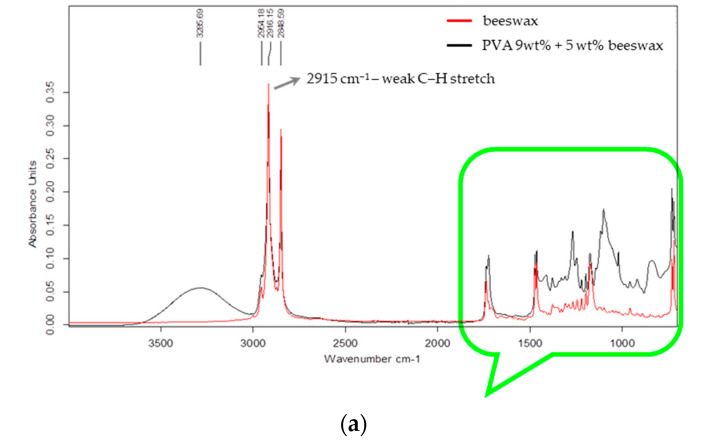

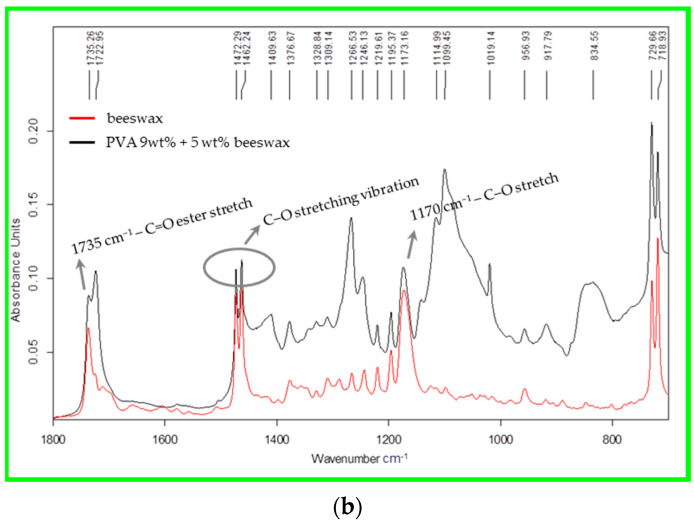
FTIR spectra of purified beeswax (red) and PVA/BW nanofibers (black): (**a**)—full spectra highlighting characteristic C–H stretching vibrations (~2800–3000 cm^−1^), (**b**)—magnified fingerprint region (700–1800 cm^−1^) showing detailed differences in functional group vibrations between beeswax and PVA/BW.

Fourier transform infrared spectroscopy (FTIR) was used to evaluate the chemical structure of the electrospun nanofibers and to assess the possible interactions between PVA and beeswax in the composite mats. The FTIR spectra of the PVA and PVA/BW nanofibers are presented in Figure 7a,b.

The spectra presented in Figure 7 illustrate two key wavenumber regions: 4000–2000 cm^−1^ (Figure 7a) and 1800–700 cm^−1^ (Figure 7b). In the spectrum of PVA/9 wt% BW, in the range 4000–2000 cm^−1^ (Figure 6a) the molecular structure associated with hydrocarbon absorption bands is dominant. A broad absorption band is observed at approximately 3287 cm^−1^, associated with the main chain of the molecule (–CH–CH (OH) –) group and intramolecular hydrogen bonds. Prominent peaks observed at 2916 and 2848 cm^−1^ correspond to asymmetric and symmetric C–H stretching vibrations of methylene groups (–CH_2_–), originating from both the PVA backbone and the long aliphatic hydrocarbon chains present in beeswax.

The main spectral differences between beeswax and the mixed PVA/BW composition are observed in the region 1800–700 cm^−1^, which are associated with the vibrations of the ester and free fatty acids (at 1735, 1722, and 1173 cm^−1^), Figure 7b. This indicates that the beeswax is physically embedded within the nanofiber structure rather than being chemically bound.

A sharp band at 1735 cm^−1^, though weak in intensity, may be associated with residual acetate groups (C=O stretching) from incomplete hydrolysis of the polymer. Both the degree of hydrolysis and the viscosity of the base PVA polymer affect the FTIR spectral features, especially in regions sensitive to functional group content and molecular mobility.

Other notable peaks include the C–H bending vibration at 1472–1462 cm^−1^ and the characteristic C–O stretching vibration of secondary alcohols at around 1088 cm^−1^ [51]. The FTIR spectrum of the PVA/BW nanofibers exhibited all characteristic peaks of PVA, with some additional or intensified bands attributable to beeswax. The carbonyl stretching band near 1735 cm^−1^ became more prominent, indicating the presence of ester linkages from wax components such as monoesters and diesters [52]. No new peaks or significant shifts in peak position were observed that would indicate chemical reactions. The overlapping of characteristic bands suggests that the two components are physically blended, with beeswax dispersed throughout the PVA matrix without chemical modification. This is evident from the change in the height of the peaks at 1173 cm^−1^ and the soil of a small peak at 956 cm^−1^. The hydrogen bonding between hydroxyl groups of PVA and polar groups in BW may contribute to minor changes in band intensity.

These findings support the interpretation that the PVA/BW nanofibers form a physically blended system, in which beeswax is entrapped or distributed within the polymer network rather than being chemically bonded.

### 3.3. Thermal Properties of PVA and PVA/BW Nanofibers

#### TGA and DSC

**Figure 8:**
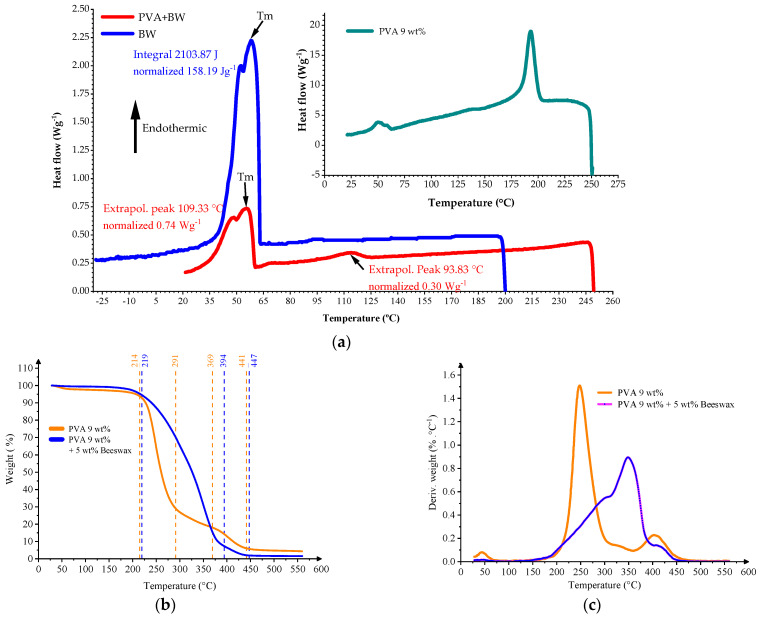
Thermal analysis of PVA and PVA/BW nanomats: (**a**) differential Scanning Calorimetry (DSC) thermograms, (**b**) thermogravimetric Analysis (TGA), (**c**) Derivative TGA (dTG) curves.

Thermal stability and transitions of the electrospun nanofibers were assessed using thermogravimetric analysis (TGA) and differential scanning calorimetry (DSC). Figure 8 presents the thermograms of the PVA and PVA/BW nanomats, while the key thermal transition data obtained from DSC analysis, including melting and crystallization parameters for all samples, are summarized in Table 2.

The TGA curves show a two-step degradation process for both materials. For PVA nanofibers, the first weight loss stage (30–100 °C) corresponds to the evaporation of physically adsorbed water. The second major decomposition phase, beginning around 230 °C and peaking near 420 °C, is attributed to thermal degradation of the polymer backbone via chain scission and elimination reactions [53].

In comparison, the PVA/BW composite nanofibers exhibited an altered thermal profile. The initial degradation peak appeared near 388 °C, and a second broader peak occurred around 454 °C. These shifts suggest that beeswax contributes to improved thermal stability. The enhanced resistance to decomposition is likely due to the wax’s long-chain hydrocarbons and ester content, which degrade more slowly and can impede thermal transport [54].

DSC analysis confirmed the TGA trends and further differentiated thermal transitions between the two materials. The DSC thermogram of PVA showed a broad endothermic peak in the 30–75 °C range, corresponding to moisture loss, followed by a sharp melting peak around 200 °C (ΔH = 5.4 J·g^−1^). A small exothermic peak near 220 °C was also observed, possibly related to recrystallization or the onset of degradation.

In the PVA/BW nanofibers, the thermal behavior was more complex. A broad melting region between 30 and 100 °C indicated the presence of beeswax components with varying melting points. The overall enthalpy of melting was significantly higher (ΔH = 53.3 J·g^−1^), consistent with the high crystalline content of BW. An additional exothermic transition near 145 °C may be attributed to recrystallization of wax esters. Similar broad and overlapping thermal transitions have been reported for beeswax-containing materials [55], reflecting the multi-component nature of the wax, including monoesters, diesters, and hydrocarbons.

These findings confirm that beeswax improves the thermal behavior of PVA nanofibers by increasing their decomposition temperature, broadening thermal transitions, and introducing new crystalline melting phases. This thermal shielding effect of beeswax has also been reported in other polymer-wax systems [56], where it delays decomposition by reducing oxygen diffusion and forming protective barriers during heating.

### 3.4. Fiber Deposition and Collector Surface Effects

**Figure 9:**
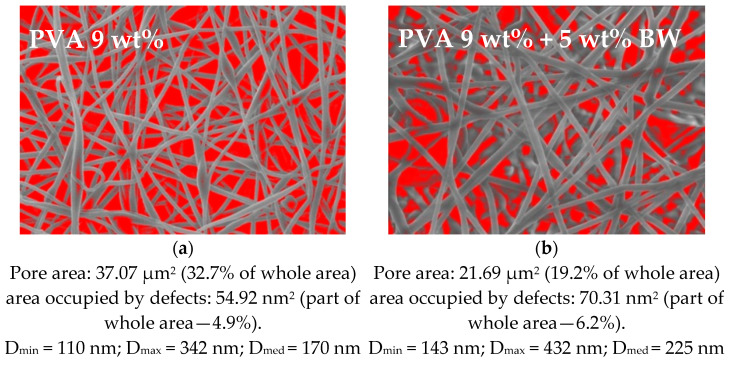
Quantitative pore area analysis of electrospun nanofiber mats using ImageJ: (**a**) PVA 9 wt%, (**b**) PVA/BW 9 wt% + 5 wt%. Red regions indicate detected pores.

**Figure 10:**
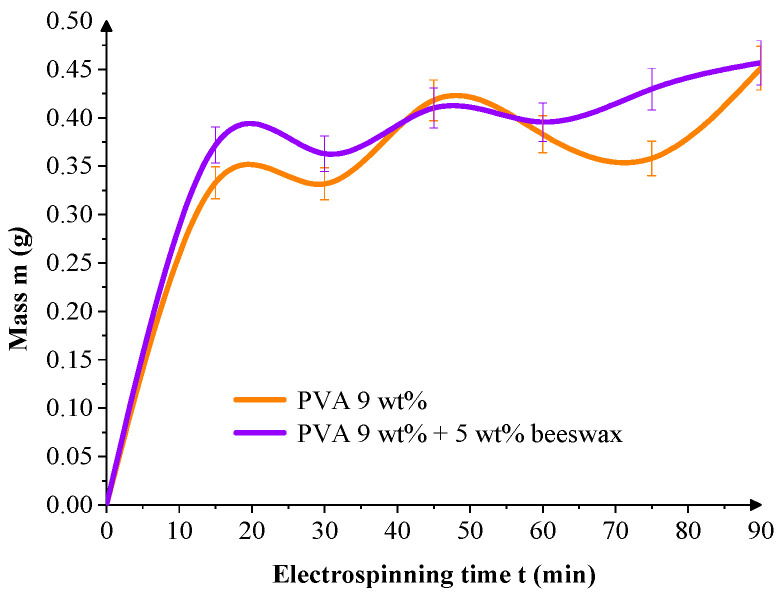
Mass accumulation on the textile collector during electrospinning of PVA and PVA/BW formulations over time. Minor oscillations in the measured values are attributed to natural jet instabilities but do not affect the overall increasing trend.

**Figure 11:**
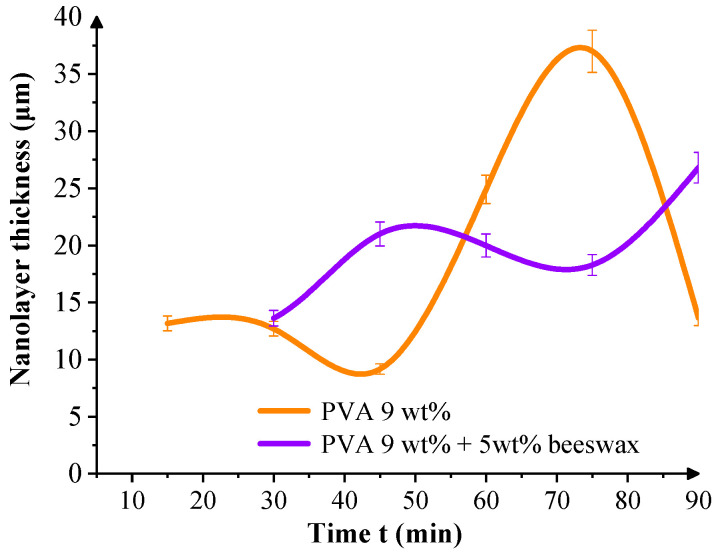
Thickness of the electrospun fiber PVAmin and PVA/BW mats measured at corresponding time intervals. The wave-like variations reflect deposition irregularities typical of dynamic electrospinning on textile substrates. Despite this, the data show a clear cumulative thickening pattern.

The nature of the collector surface plays a key role in determining fiber alignment, porosity, mat density, and deposition uniformity during electrospinning. In this study, the use of a coarse cotton fabric as a collector led to enhanced fiber retention, denser layer formation, and more uniform deposition compared to flat metallic collectors, as illustrated in Figure 9, Figure 10 and Figure 11.

As shown in Figure 9, the pore area of the PVA mat was calculated to be approximately 37.07 μm^2^, representing 32.7% of the total image area. In contrast, the PVA/BW mat showed a significantly reduced average pore area of 21.69 μm^2^, or 19.2%, indicating denser fiber packing and more compact network formation. As shown in Figure 9, the SEM micrographs highlight the relatively coarse morphology and uneven surface texture of the nanofiber mat. The altered morphology of the nanomat is likely attributed to improved inter-fiber adhesion, resulting from the partial migration of beeswax to the fiber surface, as well as changes in jet dynamics induced by its incorporation.

To evaluate deposition dynamics, the mass of collected fiber per unit area was tracked over time. Figure 10 shows that both mats exhibit rapid initial growth within the first 30 min, but PVA/BW mats demonstrated more linear and continuous accumulation over the full 90-min electrospinning period. This suggests that beeswax plays a role in the morphology of the resulting mat and enhances the stability of the deposition process, probably due to its hydrophobic nature, which influences jet-collector interactions, reduces electrostatic repulsion, and introduces plasticizing effects during fiber solidification.

Figure 11 presents the change in the thickness of the nanofibrous mat as a function of the electrospinning duration for both PVA and PVA/BW systems. In both cases, a nonlinear trend is observed, indicating that the growth of the nanomaterial does not proceed at a constant rate over time. For the PVA/BW composite, the increase in the deposited mass is accompanied by a more uniform and constant thickness profile over the collector surface. This behavior is likely due to the surface migration of the beeswax during the electrospinning process, which facilitates improved adhesion and arrangement of the fibers on the coarse cotton fabric. The hydrophobic nature of the beeswax can reduce the electrostatic repulsion and improve cohesion between the fibers of the nanomat and those on the collector surface. Similar effects have been reported in previous studies [50], where wax-containing systems showed peripheral migration of wax during solvent evaporation, leading to altered surface energy and improved mattress compaction.

The slight oscillations observed in Figure 10 and Figure 11 are attributed to natural instabilities during the electrospinning process, including fluctuations in jet trajectory, local humidity, and surface charge accumulation on the textile collector. Such variations are typical in ambient electrospinning setups. Nonetheless, the cumulative trends remain consistent, showing that both mass and thickness increase progressively with spinning time for all formulations.

The influence of the collector topography, combined with the modified physicochemical properties of its surface and the electrospinning solution, has a synergistic effect on fiber deposition. The solution containing beeswax facilitates the formation of a denser and more flexible carrier, which is consistent with previous reports that wax-modified systems show improved thermal and deposition behavior due to enhanced interfacial interactions and controlled fiber layering [56,57].

Overall, these results highlight the importance of the surface properties of the nanofibers for the adhesion between the nanofiber mat and its support when designing electrospun materials for biomedical applications where contact with complex, uneven surfaces under dynamic conditions is expected.

### 3.5. Solubility of the Nanomat in Saline

Nanocarriers used for drug delivery must be biodegradable and biocompatible. In vitro release of the active substance, in this case beeswax, is largely influenced by the solubility of the fibers on its basis. These initial solubility studies, by changing the mass of a PVA and PVA/PV mat when placed in saline, provide primary data on the influence of beeswax, which in this case is a regenerative substance acting on the skin, on the dissolution rate of the semipermeable nanofiber barrier. A physiological saline solution (0.9% NaCl) at 36 ± 1 °C was chosen as the medium for releasing beeswax from the electrospun nanofiber mats to simulate biological conditions relevant to wound dressing and regenerative applications.

**Figure 12:**
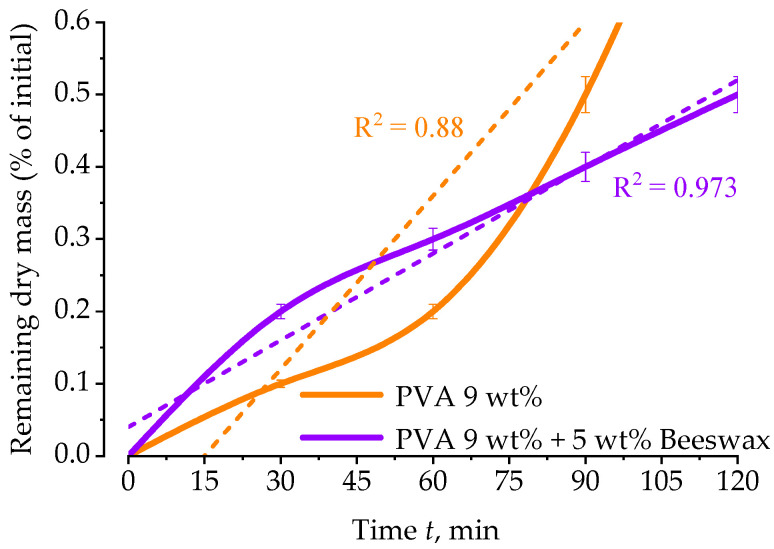
Dissolution profiles of electrospun nanofiber mats composed of polyvinyl alcohol (PVA, 9 wt%) and PVA blended with 5 wt% beeswax (PVA/BW), expressed as percentage of residual dry mass after immersion in 0.9% NaCl solution at 36 ± 1 °C.

Figure 12 depicts the mass change in electrospun nanofiber mats—composed of PVA (9 wt%) and PVA blended with 5 wt% beeswax—over time during immersion in physiological saline. The dissolution behavior reflects the influence of composition on fiber solubility and matrix integrity under simulated physiological conditions. In the presence of beeswax, the obtained nanofibers are mostly of larger diameter, the mats obtained from them have lower porosity (pore area: 21.69 μm^2^ and 19.2% of whole area) compared to those from PVA (pore area: 37.07 μm^2^ and 32.7% of whole area). The PVA layer shows a faster and nonlinear decrease in the mass of the nanofiber sample occurring within the first 30 min. By 120 min, the PVA mat had largely dissolved, confirming its high water solubility and limited resistance to ionic environments. This behavior is characteristic of the high hydrophilicity and water solubility of PVA, leading to rapid swelling and disintegration of the fibrous network. The fitted curve (R^2^ = 0.88) suggests accelerated degradation kinetics, particularly after the initial lag phase. In contrast, the PVA/BW nanofiber mat demonstrated a more controlled and linear mass change throughout the 120-min period, with a stronger correlation to first-order kinetics (R^2^ = 0.973). This indicates a more predictable and sustained dissolution profile. The incorporation of beeswax—a hydrophobic, crystalline natural additive—appears to reduce the rate of water penetration and fiber swelling, thereby enhancing matrix stability. This is further supported by previous observations of reduced porosity and increased fiber diameter in the PVA/BW mats. The presence of beeswax likely forms a partial barrier on or near the fiber surface, impeding liquid uptake and delaying structural breakdown. Different cases of chemical dissolution obey different kinetic laws [57].

The solubility behavior of the electrospun nanofiber mats was assessed by immersing pre-weighed dry samples in 0.9% NaCl solution at 36 ± 1 °C for up to 72 h. At each time point, the samples were gently removed, dried at room temperature, and re-weighed to determine the remaining dry mass. The results are presented in Figure 12 as the percentage of residual mass, calculated relative to the initial dry mass (taken as 100%). This method provides a direct measure of the material’s structural stability in aqueous environments over time.

As shown, the neat PVA nanofibers rapidly dissolved, exhibiting a sharp decrease in residual mass within the first few hours, consistent with the known water-soluble nature of low hydrolysis-degree PVA. In contrast, the PVA/BW mats retained significantly more mass throughout the study period, suggesting that beeswax incorporation reduces solubility by promoting hydrophobic interactions and potential physical crosslinking effects within the fiber network.

Notably, the pure PVA mat showed only a moderate mass loss, although low-hydrolysis PVA is generally water-soluble. This apparent stability can be rationalized by a physical network formation that occurs during electrospinning and ambient drying. The rapid solvent evaporation favors partial crystallization of PVA chains, while dense hydrogen-bonding between adjacent fibers, combined with mechanical interlocking on the textile collector, creates a quasi-cross-linked architecture. Such a physically entangled/crystalline network delays fiber disentanglement and slows dissolution, even in the absence of chemical crosslinkers. A similar pseudo-crosslinking effect has been reported for electrospun PVA systems processed under high electric fields [58].

These findings confirm that beeswax not only modifies the morphological and thermal characteristics of PVA nanofibers but also significantly improves their resistance to aqueous dissolution, making the composite system more suitable for biomedical applications requiring controlled degradation, such as wound care and drug delivery platforms.

These findings support the hypothesis that incorporating BW into PVA nanofibers not only modifies their morphology and thermal properties, but also provides functional advantages for biomedical use-particularly in moist or exudate-rich environments where controlled degradation and durability are essential.

A two-way analysis of variance (ANOVA) was conducted to examine the effects of composition (PVA vs. PVA/BW) and immersion time (30, 60, 90, and 120 min) on the percentage of mass loss during in vitro degradation in saline. The analysis showed that neither the main effect of composition (*F*(1,16) = 3.44, *p* = 0.082) nor immersion time (*F*(3,16) = 0.66, *p* = 0.587) reached statistical significance. Additionally, the interaction between composition and time was not significant (*F*(3,16) = 0.006, *p* = 0.999). Although a visual trend toward slower degradation in PVA/BW mats was observed, the difference did not reach statistical significance, likely due to the limited sample size (n = 3).

## 4. Conclusions and Outlook

This study demonstrated the successful fabrication and characterization of electro-spun nanofiber mats composed of polyvinyl alcohol (PVA) and natural beeswax (BW) for potential biomedical use. A major strength of the work lies in the development of a stable PVA/BW microemulsion suitable for electrospinning, enabling the incorporation of hydrophobic wax into a hydrophilic polymer matrix without chemical modification or surfactants.

Morphological analysis (SEM) showed that BW increased fiber diameter and surface roughness, consistent with changes in viscosity and phase interactions. FTIR confirmed the physical incorporation of BW into the PVA matrix, while TGA and DSC analyses demonstrated improved thermal stability and melting behavior, attributed to the crystal-line structure of BW. These thermal enhancements support practical advantages in processing, storage, and sterilization. Additionally, the mats exhibited denser layering and more uniform thickness, indicating improved deposition control.

Dissolution studies in saline revealed a slower, more uniform degradation profile for PVA/BW mats, consistent with enhanced water resistance. Although the observed difference was not statistically significant (*p* = 0.082, n = 3), the trend suggests increased durability in moist environments.

Despite these promising findings, some limitations remain. The lack of emulsion droplet size analysis and the small sample size for degradation studies may limit interpretability. Moreover, this study did not include biological assessments, such as cytotoxicity or antimicrobial activity, which are essential for clinical translation. Future work should also address batch-to-batch variability of natural BW by standardizing its composition.

**Figure 13:**
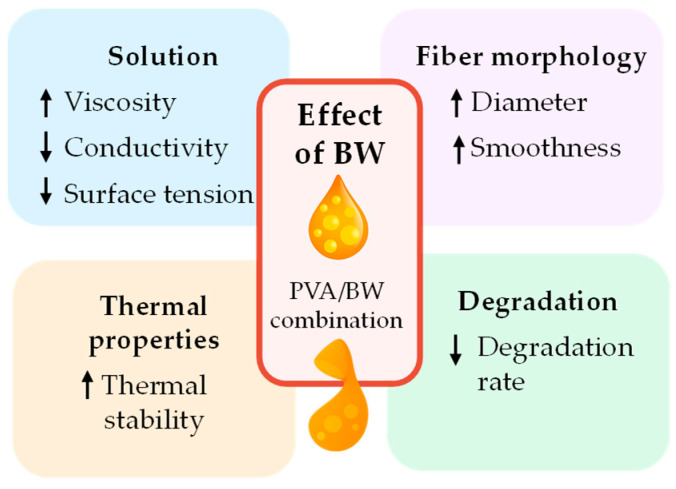
Schematic summary of the effects of beeswax (BW) incorporation into PVA electrospun nanofibers. BW influences solution behavior, spinning performance, fiber morphology, and stability under physiological conditions, contributing to the functional enhancement of the composite material.

The incorporation of 5 wt% BW had a clear impact across all levels of the system: it increased viscosity and reduced conductivity at the solution stage, altered fiber morphology in the solid state, and delayed aqueous degradation. These findings demonstrate that BW acts not only as a passive additive but as a functional component that enhances the overall performance of electrospun PVA fibers, Figure 13.

Overall, the results position the PVA/BW composite mats as promising candidates for moisture-resistant, thermally stable nanofiber scaffolds in wound care and regenerative applications. While further biological validation is required, the physicochemical improvements observed here offer a solid foundation for future biomedical exploration.

## Figures and Tables

**Table 1:** Summary of fiber diameter parameters based on SEM analysis.

Solution Type	Mean Diameter (nm)	Standard Deviation (SD)	Min Diameter Value (nm)	Max Diameter Value (nm)
PVA9 wt%	170	0.043	110	342
PVA9 wt% + 5 wt% BW	225	0.055	143	432

**Table 2:** DSC thermal properties of PVA, beeswax, and PVA/BW samples.

Sample Label	Melting Temperature Tm Onset (°C)	Melting Temperature Tm Peak (°C)	Melting Enthalpy (J·g^−1^)	Crystallization Temperature Tc Onset (°C)	Crystallization Temperature Tc Peak (°C)	Crystallization Enthalpy (J·g^−1^)
Beeswax	45.4	66.5	162.4	62.8	58.0	165.0
PVA 9 wt%	34.2	50.0	31.0	-	-	-
PVA9 wt% + BW5 wt%	36.8	62.5	53.3	-	-	-

## Data Availability

The original contributions presented in the study are included in the article, further inquiries can be directed to the corresponding author.

## References

[1] Bonakdar M.A., Rodrigue D. (2024). Electrospinning: Processes, Structures, and Materials. Macromol.

[2] Bölgen N., Demir D., Aşık M., Sakım B., Vaseashta A., Vaseashta A., Bölgen N. (2022). Introduction and Fundamentals of Electrospinning. Electrospun Nanofibers: Principles, Technology and Novel Applications.

[3] Figen A.K. (2020). History, Basics, and Parameters of Electrospinning Technique. Electrospun Materials and Their Allied Applications.

[4] Neznakomova M.P., Salaün F., Gospodinova D.N. Preparation and Characterization of Electrospun Nutraceptic Nanofibers from Polyvinyl Alcohol/Beeswax. Proceedings of the 2023 International Scientific Conference on Computer Science (COMSCI).

[5] Xue J., Wu T., Dai Y., Xia Y. (2019). Electrospinning and Electrospun Nanofibers: Methods, Materials, and Applications. Chem. Rev..

[6] Kopp A., Smeets R., Gosau M., Kröger N., Fuest S., Köpf M., Kruse M., Krieger J., Rutkowski R., Henningsen A. (2020). Effect of process parameters on additive-free electrospinning of regenerated silk fibroin nonwovens. Bioact. Mater..

[7] Anjum S., Rahman F., Pandey P., Arya D.K., Alam M., Rajinikanth P.S., Ao Q. (2022). Electrospun Biomimetic Nanofibrous Scaffolds: A Promising Prospect for Bone Tissue Engineering and Regenerative Medicine. Int. J. Mol. Sci..

[8] Vitchuli N., Shi Q., Nowak J., McCord M., Bourham M., Zhang X. (2010). Electrospun ultrathin nylon fibers for protective applications. J. Appl. Polym. Sci..

[9] Huang C., Xu X., Fu J., Yu D.-G., Liu Y. (2022). Recent Progress in Electrospun Polyacrylonitrile Nanofiber-Based Wound Dressing. Polymers.

[10] Kotni T.R., Pandey S., Shekhar S., Ranjan R., Srivastava P.S. (2023). Synthesis of PVA nano fibers by using electrospinning. Mater. Today Proc..

[11] Bognitzki M., Hou H., Ishaque M., Frese T., Hellwig M., Schwarte C., Schaper A., Wendorff J.H., Greiner A. (2000). Polymer, Metal, and Hybrid Nano- and Mesotubes by Coating Degradable Polymer Template Fibers (TUFT Process). Adv. Mater..

[12] Fan T., Qin J., Meng X., Li J., Liu Q., Wang G. (2022). Biodegradable membrane of poly(l-lactide acid-dioxanone-glycolide) and stereocomplex poly(lactide) with enhanced crystallization and biocompatibility. Front. Bioeng. Biotechnol..

[13] Flores-Rojas G.G., Gómez-Lazaro B., López-Saucedo F., Vera-Graziano R., Bucio E., Mendizábal E. (2023). Electrospun Scaffolds for Tissue Engineering: A Review. Macromol.

[14] Nguyen T.D., Roh S., Nguyen M.T.N., Lee J.S. (2023). Structural Control of Nanofibers According to Electrospinning Process Conditions and Their Applications. Micromachines.

[15] Zulkifli M.Z.A., Nordin D., Shaari N., Kamarudin S.K. (2023). Overview of Electrospinning for Tissue Engineering Applications. Polymers.

[16] Chen L., Wang S., Yu Q., Topham P.D., Chen C., Wang L. (2019). A comprehensive review of electrospinning block copolymers. Soft Matter.

[17] Yang J., Hu J. (2023). Electrospun Polyurethanes. Polyurethanes: Preparation, Properties, and Applications Volume 2: Advanced Applications.

[18] Al-Abduljabbar A., Farooq I. (2023). Electrospun Polymer Nanofibers: Processing, Properties, and Applications. Polymers.

[19] Maran B.A.V., Jeyachandran S., Kimura M. (2024). A Review on the Electrospinning of Polymer Nanofibers and Its Biomedical Applications. J. Compos. Sci..

[20] Kyuchyuk S., Paneva D., Karashanova D., Markova N., Georgieva A., Toshkova R., Manolova N., Rashkov I. (2022). Core-Sheath-Like Poly(Ethylene Oxide)/Beeswax Composite Fibers Prepared by Single-Spinneret Electrospinning. Antibacterial, Antifungal, and Antitumor Activities. Macromol. Biosci..

[21] Kyuchyuk S., Paneva D., Manolova N., Rashkov I., Karashanova D., Naydenov M., Markova N. (2023). Electrospun Fibers of Biocompatible and Biodegradable Polyesters, Poly(Ethylene Oxide) and Beeswax with Anti-Bacterial and Anti-Fungal Activities. Materials.

[22] Blachowicz T., Ehrmann A. (2020). Conductive Electrospun Nanofiber Mats. Materials.

[23] Buchko C.J., Kozloff K.M., Martin D.C. (2001). Surface characterization of porous, biocompatible protein polymer thin films. Biomaterials.

[24] Chernonosova V., Khlebnikova M., Popova V., Starostina E., Kiseleva E., Chelobanov B., Kvon R., Dmitrienko E., Laktionov P. (2023). Electrospun Scaffolds Enriched with Nanoparticle-Associated DNA: General Properties, DNA Release and Cell Transfection. Polymers.

[25] Soukarie D., Nocete L., Bittner A.M., Santiago I. (2024). DNA data storage in electrospun and melt-electrowritten composite nucleic acid-polymer fibers. Mater. Today Bio.

[26] Biagiotti M., Bassani G.A., Chiarini A., Vincoli V.T., Dal Prà I., Cosentino C., Alessandrino A., Taddei P., Freddi G. (2022). Electrospun Silk Fibroin Scaffolds for Tissue Regeneration: Chemical, Structural, and Toxicological Implications of the Formic Acid-Silk Fibroin Interaction. Front. Bioeng. Biotechnol..

[27] Habibzadeh F., Sadraei S.M., Mansoori R., Chauhan N.P.S., Sargazi G. (2022). Nanomaterials supported by polymers for tissue engineering applications: A review. Heliyon.

[28] Kidoaki S., Kwon I.K., Matsuda T. (2005). Mesoscopic spatial designs of nano- and microfiber meshes for tissue-engineering matrix and scaffold based on newly devised multilayering and mixing electrospinning techniques. Biomaterials.

[29] Gaaz T.S., Sulong A.B., Akhtar M.N., Kadhum A.A.H., Mohamad A.B., Al-Amiery A.A. (2015). Properties and Applications of Polyvinyl Alcohol, Halloysite Nanotubes and Their Nanocomposites. Molecules.

[30] Özekmekci M., Ünlü D., Çopur M. (2021). PVA/Amberlit IRA 743 Hibrit Membran İle Endüstriyel Atık Sudan Bor Giderimi. https://hdl.handle.net/20.500.12885/1990.

[31] Park J.C., Ito T., Kim K.O., Kim K.W., Kim B.S., Khil M.S., Kim H.Y., Kim I.S. (2010). Electrospun poly(vinyl alcohol) nanofibers: Effects of degree of hydrolysis and enhanced water stability. Polym. J..

[32] Hamilton R.J. (1978). Chemistry and Biochemistry of Natural Waxes. Biochem. Soc. Trans..

[33] Patel S., Nelson D.R., Gibbs A.G. (2001). Chemical and physical analyses of wax ester properties. J. Insect Sci..

[34] Liu D., Duan Y., Wang S., Gong M., Dai H. (2022). Improvement of Oil and Water Barrier Properties of Food Packaging Paper by Coating with Microcrystalline Wax Emulsion. Polymers.

[35] Trinh B.M., Smith M., Mekonnen T.H. (2022). A nanomaterial-stabilized starch-beeswax Pickering emulsion coating to extend produce shelf-life. Chem. Eng. J..

[36] Zhang Y., Simpson B.K., Dumont M.-J. (2018). Effect of beeswax and carnauba wax addition on properties of gelatin films: A comparative study. Food Biosci..

[37] Brito-Pereira R., Ribeiro C., Tubio C.R., Castro N., Costa P., Lanceros-Mendez S. (2023). Beeswax multifunctional composites with thermal-healing capability and recyclability. Chem. Eng. J..

[38] Dobrosielska M., Dobrucka R., Kozera P., Brząkalski D., Gabriel E., Głowacka J., Jałbrzykowski M., Kurzydłowski K.J., Przekop R.E. (2023). Beeswax as a natural alternative to synthetic waxes for fabrication of PLA/diatomaceous earth composites. Sci. Rep..

[39] Sowmya B., Panda P.K. (2023). Electrospun poly (ε-caprolactone)/beeswax based super-hydrophobic anti-adhesive nanofibers as physical barriers for impeding fibroblasts invasion. J. Biomater. Appl..

[40] Zou F., Tan C., Shinali T.S., Zhang B., Zhang L., Han Z., Shang N. (2023). Plant antimicrobial peptides: A comprehensive review of their classification, production, mode of action, functions, applications, and challenges. Food Funct..

[41] Ahn K., Kambiz S., Park K., Seo J. (2024). Enhancing polyvinyl alcohol surface properties through pre-drying treatment and modified electrospun polycaprolactone nanofibers. Prog. Org. Coat..

[42] Szumała P., Luty N. (2016). Effect of different crystalline structures on W/O and O/W/O wax emulsion stability. Colloids Surf. Physicochem. Eng. Asp..

[43] Bogdanov S. (1997). Nature and Origin of the Antibacterial Substances in Honey. LWT-Food Sci. Technol..

[44] Lazarov S., Veleva P., Zhelyazkova I. (2022). Physicochemical characteristics of Bulgarian bee honey: Part 1. Bulg. J. Agric. Sci..

[45] Filatov Y. *Elektroformovanie Voloknistykh Materialov*, Monograhy, Neft and Gas: Moscow, Russia, 2001. https://scholar.google.com/scholar_lookup?&title=Elektroformovanie%20voloknistykh%20materialov&publication_year=2001&author=Filatov%2CYu.N.

[46] Nayak P., Ghosh A.K., Bhatnagar N. (2022). Investigation of Solution Rheology in Electrospinning of Ultra High Molecular Weight Polyethylene. Fibers Polym..

[47] Hotaling N.A., Bharti K., Kriel H., Simon C.G. (2015). DiameterJ: A validated open source nanofiber diameter measurement tool. Biomaterials.

[48] Stanger J.J., Tucker N., Buunk N., Truong Y.B. (2014). A comparison of automated and manual techniques for measurement of electrospun fibre diameter. Polym. Test..

[49] Rebia R.A., Sadon N.S.B., Tanaka T. (2019). Natural Antibacterial Reagents (Centella, Propolis, and Hinokitiol) Loaded into Poly[(R)-3-hydroxybutyrate-co-(R)-3-hydroxyhexanoate] Composite Nanofibers for Biomedical Applications. Nanomaterials.

[50] Razavizadeh B.M., Niazmand R. (2020). Characterization of polyamide-6/ propolis blended electrospun fibers. Heliyon.

[51] Svečnjak L., Baranović G., Vinceković M., Prđun S., Bubalo D., Gajger I.T. (2015). An Approach for Routine Analytical Detection of Beeswax Adulteration Using FTIR-ATR Spectroscopy. J. Apic. Sci..

[52] Wu X., Wang Z., Teng J., Yang L., Xu S., Luo S., Wu Z., Ye C. (2025). Electrospun microfiber composite scaffolds of polyvinyl alcohol, polyhydroxybutyrate, and multiwalled carbon nanotubes for enhancing the osteogenic differentiation of stem cells to promote bone regeneration. Int. J. Biol. Macromol..

[53] Alexy P., Káchová D., Kršiak M., Bakoš D., Šimková B. (2002). Poly(vinyl alcohol) stabilisation in thermoplastic processing. Polym. Degrad. Stab..

[54] Tran N.H.A., Brünig H., Hinüber C., Heinrich G. (2014). Melt Spinning of Biodegradable Nanofibrillary Structures from Poly(lactic acid) and Poly(vinyl alcohol) Blends. Macromol. Mater. Eng..

[55] Buchwald R., Breed M.D., Greenberg A.R. (2008). The thermal properties of beeswaxes: Unexpected findings. J. Exp. Biol..

[56] Schuman Y. Thermal Analysis of Phase Change Materials—Three Organic Waxes Using TGA, DSC, and Modulated DSC^®^—TA Instruments. TA Instruments, TA405. https://www.tainstruments.com/applications-notes/thermal-analysis-of-phase-change-materials-three-organic-waxes-using-tga-dsc-and-modulated-dsc/.

[57] Ritger P.L., Peppas N.A. (1987). A simple equation for description of solute release I. Fickian and non-fickian release from non-swellable devices in the form of slabs, spheres, cylinders or discs. J. Control. Release.

[58] Enayati M.S., Behzad T., Sajkiewicz P., Bagheri R., Ghasemi-Mobarakeh L., Łojkowski W., Pahlevanneshan Z., Ahmadi M. (2016). Crystallinity Study of Electrospun Poly (Vinyl Alcohol) Nanofibers: Effect of Electrospinning, Filler Incorporation, and Heat Treatment. Iran. Polym. J..

